# Report of the Committee Appointed on the Occasion of the Death of Dr. Bryant Burwell

**Published:** 1862-02

**Authors:** 


### EDITORIAL DEPARTMENT.

### REPORT OF THE COMMITTEE APPOINTED ON THE OCCASION OF THE DEATH OF DR. BRYANT BURWELL.

At a special meeting of the Erie County Medical Society, convened for the purpose of taking such action as might seem proper upon the death of Dr. Bryant Burwell, one of the oldest and most respected members of the Society; it was

*Resolved,*—That a committee consisting of Drs. Josiah Trowbridge, Moses Bristol, A. S. Sprague, Gorham F. Pratt, Josiah Barnes and Charles Winne, report at the next regular meeting of the Erie County Medical Society.

In compliance with this action, your committee respectfully submit the following report and resolutions:

"The committee have obtained from the sources to which it has had access the following sketch of the life and professional history of our lamented colleague.

"It is a remark as true as it is old that the life and works of the most eminent physicians are known and appreciated by a very limited number of their cotemporaries. Their abstracted and almost isolated position in society arises from the very nature of their avocation. In the earlier portion of their career, they exist only as ciphers in the multitude striving for position, wealth and power.

"The humble and obscure medical man is not called to lend his aid to the movements of the popular masses engaged in the political agitations of the day, when men are animated by the lust for fame and exalted position.—Nor in the busy marts of commerce, where wealth allures and in the distance are seen as its attendants, leisure, luxury, place and power. By the physician also, the toils and sacrifices of war are to be borne, although to him all of the glory that gilds its terrific and bloody character is denied; his arena is the chamber of the sick, the bed of suffering and of death; his daily and hourly intercourse is with the querulous and dissatisfied invalid; he is animated by no incentive of glittering rewards, by no popular applause, no extended reputation; his life is passed with little of the charm of pleasant intercourse with friends and a very limited assocation with his professional brethren; thus time rolls on, and in the vigor of youth, when with a mind richly stored with recorded experience, and again when in the prime of life still richer with his own personal acquisitions and practical skill, he is doomed to wait until hope sickens for that consideration from the public, to which he justly looked; and it is often, only when youth is gone and manhood is waning and the infirmities of age are approaching, that he is properly appreciated.

"The life of our lamented colleague, fortunately for himself and the community in which he resided and passed the greater part of his professional career, can not be regarded as marked by the extreme features, which we have in our exordium accorded to the larger part we fear of practitioners of medicine in our large cities and the more populous portions of our country. Doctor Burwell was by nature endowed with a firm and vigorous constitution, that was developed and strengthened by habits of exercise and labor pertaining to country life, conjoining to a sound body that healthy play of the mental functions, that of itself is so powerful in determining the happiness and usefulness of its possessors. To these early habits of industry and exercise in a healthful atmosphere, was in a great measure due his ability to undergo the great amount of physical exposure and fatigue to which he was subjected in his extensive practice. There are but few of us who have not often expressed our admiration of his vigorous constitution, his commanding and dignified personal appearance, united as these were to a captivating address, a temper seldom ruffled, and a benignity of deportment rarely equalled.

"Your committee believe that to many members of this society, the deceased was as well, perhaps better known than to us, and we have not a doubt, that all of his professional brethren will agree with us, that we have given a truthful account of his personal attributes.

"The subject of this memoir, as we learn from Doctor G. N. Burwell, the son of the deceased, was born in the town of Russia, Herkimer county, state of New York, August 26, 1796; that he early emerged from the common schools of that period to become himself a teacher of youth. Then commenced his more active and studious career of self education. With the text books he passed through the ordinary English academical course, and with access to the village library, he made himself acquainted with general history, literature and the British classics.

"In 1814, when eighteen years of age, he entered the medical office of the eminent Professor Willoughby in the village of Newport, adjoining his native town; his kind friend Dr. Jacob L. Sherwood, was then a partner with Professor Willoughby. After attending two courses of lectures at the Fairfield Medical College, given by the gifted Professors Willoughby, Hadley, T. Romeyn Beck and Delamater, he married Anna Clark, of Newport, and commenced as a licentiate of medicine the practice of his profession, in the adjacent village and town of Norway under the generous auspices of his preceptors Willoughby and Sherwood. His promptitude, geniality and success, attached him as a friend to that community, which deeply regretted his departure to the village of Buffalo in 1824. He attended the lecture course of 1822 and '23, at the Fairfield Medical College and received the degree of Doctor of Medicine. In Buffalo he first practiced in company with Doctor Cyrenius Chapin; he spent the winter of 1826 and '27, in Philadelphia, where he enjoyed the advantages of the medical schools and association with the eminent medical men of that city. Doctor B. loved his profession and was devoted to it. Everything had to yield to his paramount attention to his patients.

"The poor, who were the most numerous, always obtained as careful attendance and thought and as true sympathy as did the more able and wealthy; and beside furnishing them advice and medicine, there were at all times those whom he supplied with food and other necessaries of life; like the kind hearted Doctor Chapin, with whom he was first associated, he was never known to neglect the call of any one, and would turn out in the night, storm and tempest, to search out his poor and suffering patient, leaving his tired horse to enjoy the stable. No wonder that he was endeared to so many persons and families; that so many remember him with affection and gratitude. His relations with his brethern in the profession were truly cordial and happy; the younger ones looked up to him with confidence as a true friend, who would promote their welfare; the elder relied upon his courtesy and respect; all esteemed him as a shining example of professional etiquette. He entertained a high opinion of the physicians of Buffalo as a class, and felt honored by their association, and from this honest feeling sprang his good understanding with them. No considerable fortunes have been made in Buffalo by the medical profession and Doctor Burwell was no exception to the rule.

"This true and appreciative narrative, from the pen of one who honors himself in honoring his father, will meet the undivided commendation of all who were acquainted with the subject of this memoir. To those who succeed,—the cotemporaries and friends of Dr. B.—it will be interesting to know that solid worth and shining attainments in our profession, have not been wholly disregarded by the present members of this society; nor have we overlooked the private and domestic virtues of the deceased, nor are they yet forgotten by those who have been charmed and benefitted by them.

"In the year 1838, he was deprived by death of the society of his amiable and exemplary consort: after much patient suffering, Mrs. B., passed from life, leaving the survivor a sincere mourner and the sole protector of three children, of whom Dr. G. N. Burwell is the eldest. The youngest of the two daughters was at the time of her mother's death a delicate and sickly babe, whose life hung upon so slight a tenure, that it was feared she would not long survive her mother, It was under these afflictive circumstances, that the brightest traits that adorn human nature were eminently displayed.—Notwithstanding the cares and labors of a large practice, he gave unwearied attention to his youthful charge, and he was daily seen pursuing the duties of his profession, with his child seated in the carriage with him, to insure her air and exercise, and caring for her with all the assiduity of a nurse and the tenderness of a mother. At length his faithful supervision was rewarded by her gradual restoration to health, and he felt in his sickness and helplessness abundantly repaid by her kind offices and devoted attentions. In the year 1845, he married the widow of Joseph Clary, Esq., one of the earliest, most energetic and influential citizens of Buffalo, who had held offices of trust and honor in public life and died universally esteemed. It was the good fortune of Mrs Burwell, who survives the subject of this narrative, to have united her fate with his, when he was in the full vigor of his usefulness, in the height of his profesisonal fame. She lives to attest to all the dignity of his character and the breadth and depth of his affectionate nature. In her bereavement she is sustained, by the reflection that to her genial participation in his daily pursuits, and his love of hospitality, she contributed largely to his happiness while he could enjoy the society of his friends, and that in his sickness and sorrow she was to him, we may well say, a ministering spirit, ready to relieve his physical anguish and to solace his grief, when his strong mind was sad and heavy, by joining with him in those christian exercises which alone support man when the strength fails and life itself becomes a burden.

" At the period of their marriage, he had resolved to curtail the extent of his professional business, from which he could legitimately be relieved by his son and successor; and although at this period and for some years subsequent he was released from the hourly duty of attending to the wants of his patients, yet he was far from yielding to a slothful indulgence. His active mind was occupied in poring over the pages of history, the study of nature in all her attractive aspects, and the literature of the day, embodying the poetic and imaginative productions of the authors of this era. Nature had formed the mind of Dr. Burwell in a mould truly imaginative as well as philosophic. If to true poetry belong both the realms of fancy and truth, it may be fairly claimed that he possessed the poetic element in a high degree. If not a poet in its lofty creative power and energy, he possessed at least its highest perceptive and critical elements.

" We are informed that for a long period, he was in the habit of journalizing daily, interesting incidents in professonal life, important cases occurring in his practice and inconsultations with his brethren, and that among these daily memoranda, there breathes a spirit of kindness to his associates, and of indulgence for any want of harmony or misunderstanding that may have occurred between himself and any one of them. No word of bitterness or reproach sullies its pages, and those who loved him best, can point to it as an ever living eulogium upon his character.

"Dr. Burwell at all times took a deep interest and a very active part in all the meetings and proceedings of our National, State and County Medical Societies and Associations; his efforts were unceasingly directed both to advance the science and promote the best interests of the profession in all its relations to the public. Essential changes were at one time contemplated and discussed in both our State and County societies and halls of Legislation in regard to the alteration and amendment of the laws regulating the practice of medicine.

" Under the pressure of public opinion brought to bear upon the medical laws by demagogues and empirics a repeal of that portion, affixing penalties upon irregular practitioners, was carried through, and an abrogation of all restrictions upon the practice was eventually effected. During this agitation Dr. B. took an active and influential part. While at first it was deemed possible to preserve the conservative features of the old law, he was diligent and active in furnishing facts and arguments to the membersof the Legislature. Towards the last of this agitation it is believed he changed his views, from the circumstance that concentrated and organised opposition of physicians as a class exasperated the controversy. In the result little remained to county societies but the privilege of electing their own members and an immunity from criminal prosecution for mal-practice instigated by the ignorant or malevolent.

"The late years of Dr. B., were still marked by a diligent attendance upon the regular meetings of this society, where he always rejoiced to see and hold intercourse with his professional brethren, whom distance prevented him from otherwise seeing, and he often availed himself of the presence in the city of any eminent medical acquaintance or friend to entertain him at his hospitable mansion. Several years passed away in this agreeable round of professional, scientific and social enjoyment, and it may be truly said, that blest with a happy home, with a moderate, yet ample fortune, with children that loved and reverenced him, with numerous intellectual friends, his time passed calmly and each day promised more happiness.

"But the sad reverse of this picture was soon to be presented, and the future obscured by sickness, sorrow and irremediable gloom. Before Dr. B., was seized with the disease that terminated his life, he had been occasionally attacked with painful affections of the bowels,that impaired his strength and to recover this, he journeyed at various times.

"While in Washington he had violent lumbago, and for many months felt the effects upon his former vigorous frame. After his return, he restricted his practice and endeavored by great care to renovate his impaired condition, In the autumn of 1856, while in the house of a patient, he experienced a fullness of the head and confusion that was so severe, that he hastened to return to his dwelling; here in a short time was superadded a numbness of his arm and fingers. Medical aid was summoned; his son was soon in attendance and by the application of remedial means he was relieved. During the years that followed, he had repeated attacks of a similar nature and he felt and appreciated the warning, that his former robust health had left him, never to return.

"From the earliest recognition of these alarming symptoms, he told his attending physicians that he had no hope of restoring his shattered frame; he conversed calmly upon the dreadful reality and expressed the wish that he might bear this great calamity with christian resignation and fortitude, and if it was the will of Divine Providence that his end was approaching, he fervently prayed that his mind might be preserved, to save his family and friends the painful spectacle, of seeing his mental light go out, before his body was gathered to the dust. Throughout his protracted illness, he preserved the same considerate care for those around him and the same affectionate interest in their future welfare.

" From childhood he had taken a deep interest in agricultural pursuits and he embraced an early opportunity after his illness to retire into the country and here his last days were passed in the quiet enjoyment of the unexciting and pleasing scenes of nature. Sad as was his afflicted condition there was much to console his family, who devoted their entire thoughts and time to cheer and comfort him. Thus his weary and monotonous days weer lightened of half their tedium and gloom, and at length he passed from life with but little physical suffering, with a mind entirely reconciled to the great change that was impending, and a firm reliance upon that God, whom through life, he had worshipped in sincerity and truth.

" If, in recording this narrative of the life of Dr. Burwell, we have made it brief and uninteresting, it is not through lack of its intrinsic interest, but from our own inability to do justice to the subject, It seems to us eminently fitting in this instance of mortality, that proper notice should be taken of it by this Society and therefore we respectfully recommend the adoption of the following resolutions to be recorded in our proceedings and published in the *Buffalo Medical Journal*, and a copy of them transmitted to the State Medical Society, with a request that they be published in the transactions for 1862,

" *Whereas*—Death has deprived our society of our esteemed friend and colleague, Dr. Bryant Burwell, and whereas it is proper to give expression to our respect for the deceased: Therefore,

" *Resolved*—That in the demise of Dr. Burwell, this society has lost one of its most useful members and the community one of its brightest ornaments.

" *Resolved*—That in all the relations of professional and private life he was a model for imitation alike distinguished for his urbanity of deportment, his untiring industry, his devotion to his profession, his amiable disposition and his liberality to the poor.

" *Resolved*—That we tender to his family an expression of our warmest sympathy in their deep affliction."

